# How to simply and efficiently screen microbial strains capable of anaerobic biosynthesis of biosurfactants: Method establishment, influencing factors and application example evaluation

**DOI:** 10.3389/fmicb.2022.989998

**Published:** 2022-09-12

**Authors:** Feng Zhao, Yujing Wang, Xin Hu, Xinyu Huang

**Affiliations:** School of Life Sciences, Qufu Normal University, Qufu, China

**Keywords:** biosurfactants, anoxic conditions, environment, microplate assays, oil displacement circle, surface tension

## Abstract

Microbial resources capable of anaerobic biosynthesis of biosurfactants are increasingly interested for their application in oxygen-deficient environments, such as *in-situ* microbial enhanced oil recovery and anaerobic bioremediation. How to simply and efficiently screen microbial strains capable of anaerobic biosynthesis of biosurfactants need be further studied in depth. In this study, an efficient and simple screening method was established based on the oil displacement characteristic of biosurfactants combined with the anaerobic culture technology using microplate assays. Strains whose anaerobic culture in microwells can form oil displacement circles with diameters larger than 10 mm were screened for scale-up culture in anaerobic tubes. The screened strains which can reduce the surface tension of anaerobic culture to lower than 45 mN/m were verified as positive strains. Using this screening method, eight positive strains and thirteen positive strains were screened from oil reservoir produced water and oily sludge, respectively. Through phylogenetic analysis, some screened strains were identified as *Pseudomonas* sp., *Bacillus* sp., and *Enterobacter* sp. This study also found that more microbial strains might be isolated after enrichment culture of environmental samples, whereas more microbial species would be isolated without enrichment. Suspension of environmental samples prepared with distilled water or normal saline had no significant effect. The established screening method is highly targeted and efficient for microbial strains capable of anaerobic biosynthesis of biosurfactants. The diameter of oil displacement circle is a reliable screening indicator. This study will contribute to explore more microbial resources which can anaerobically biosynthesize biosurfactants.

## Introduction

Biosurfactants are surface active secondary metabolites generally biosynthesized by some bacteria, yeast or fungi, including lipopeptides, glycolipids, phospholipids, long-chain hydroxyl fatty acid, glycoproteins and heteropolysaccharides (Fenibo et al., 2019; Markande et al., 2021). With the increasing awareness of environmental protection, more and more attention has been paid to microbial biosurfactants (Badmus et al., 2021). Compared with chemical surfactants, biosurfactants have the advantages of high activity, low toxicity or non-toxicity, biodegradability and ecological friendliness (Phulpoto et al., 2020). Therefore, biosurfactants were extensively studied and applied in various fields such as petroleum exploitation, environmental remediation and agriculture (Fenibo et al., 2019; Markande et al., 2021; Sarubbo et al., 2022).

Most of biosurfactants producing bacteria reported in current studies are aerobic bacteria. With the expansion of biosurfactants applications, there is growing interest toward microbial resources capable of anaerobic biosynthesis of biosurfactants for their application in anoxic environments, such as oil reservoirs, the polluted sediment, deep soil and water (Domingues et al., 2017; Li et al., 2021). Microbial *in-situ* production of biosurfactants in anoxic oil reservoirs is significant to enhance oil recovery (Youssef et al., 2013; Zhao et al., 2018, 2021). Microbial *in-situ* production of biosurfactants in polluted anoxic environments can improve the anaerobic bioremediation process (Domingues et al., 2017).

However, the application of biosurfactants production in anoxic environments is still a challenge, because the microbial resources which can anaerobically biosynthesize biosurfactants are relatively scarce (Domingues et al., 2017; Li et al., 2021). More microorganisms capable of anaerobic biosynthesis of biosurfactants are urgently needed. Previous studies have reported that some strains isolated from natural environments can produce biosurfactants under anaerobic conditions such as *Bacillus licheniformis, B. subtilis, B. mojavensi, Pseudomonas* sp. and *P. aeruginosa* (Javaheri et al., 1985; Soudmand-asli et al., 2007; Albino and Nambi, 2010; Ghojavand et al., 2011; Youssef et al., 2013; Zhao et al., 2015a, 2021). These studies confirmed the feasibility of screening the microorganisms which can anaerobically biosynthesize biosurfactants from the natural environments. In oil reservoirs, sediment and other anoxic environments, the anoxic conditions resulted in the inner microbial communities with unique metabolic function (Zhao et al., 2021; Pavlova et al., 2022). Due to the nutrients chemotaxis and the evolutionary adaptation to hydrophobic substrates, there exist microorganisms capable of metabolizing petroleum hydrocarbons in oil reservoirs, oily sludge and the oil-polluted soil and water (Rodríguez et al., 2021; Zhao et al., 2021; Pavlova et al., 2022). And most of these microorganisms can produce biosurfactants to assist the biodegradation petroleum hydrocarbons through emulsifying and solubilizing petroleum hydrocarbons (Patowary et al., 2017; Phetcharat et al., 2019; Atakpa et al., 2022). These environments provide the possibility to screen microbial strains capable of anaerobic biosynthesis of biosurfactants.

How to simply and efficiently screen microbial strains capable of anaerobic biosynthesis of biosurfactants? There is no better screening method for microbial resources capable of anaerobic biosynthesis of biosurfactants. The traditional methods are not only inefficient, time-consuming and laborious, but also have limited screening flux. In the present work, an efficient and simple screening method was established based on the oil displacement characteristic of biosurfactants combined with the anaerobic culture technology using microplate assays. Two kinds of environmental samples, oil reservoir produced water and oily sludge, were used to evaluate this newly established screening method. Some screened strains were identified by phylogenetic analysis. The potential influencing factors of the established screening method were also analyzed. This method can improve screening efficiency and screening flux. This method contributes to explore more microbial resources which can anaerobically biosynthesize biosurfactants. This study will boost the applications of biosurfactants production in anoxic environments.

## Materials and methods

### Culture medium and analytical methods

The sample dilution spread plate method was used for single colony isolation. Luria–Bertani (LB) medium contained 18 g/l of agar powder was used to prepare solid plate medium. LB medium contained 10 g/l Tryptone, 5 g/l Yeast Extract, 10 g/l NaCl. The pH of medium was adjusted to 7.0. After autoclaving (121°C, 20 min), 30 ml of LB medium at 50–60°C were poured into plates. The fermentation medium used for biosurfactants production contained 30 g/l glycerol, 10 g/l glucose, 4.0 g/l NaNO_3_, 3.0 g/l KH_2_PO_4_, 4.0 g/l K_2_HPO_4_·3H_2_O, 0.80 g/l MgSO_4_·7H_2_O, CuCl_2_ 0.1 g/l, CaCl_2_·2H_2_O 0.10 g/l, ZnCl_2_ 0.10 g/l, MnCl_2_·4H_2_O 0.10 g/l. The chemicals used in this study were all with analytical grade. The diameters of oil displacement circles formed by culture broth were measured by oil spreading method. The oil spreading method was performed as previously described (Zhao et al., 2016). The crude oil used in this study was sampled from Xinjiang oilfield, China, with viscosity lower than 10 mPa·s. During the oil displacement experiments, 30 ml distilled water, 20 μl crude oil and 10 μl anaerobic culture supernatant were used. After centrifugation (8,000 *g*, 10 min), the surface tension of anaerobic culture supernatant (10 ml) was measured at 28°C using the BZY-1 surface tensiometer (Shanghai equitable Instruments Factory, Shanghai, china). Each sample was determined three times and the average value was taken.

### Establishment of a screening method using previous microbial strains

Using the previously obtained microbial strains, a method was established for screening microbial strains capable of anaerobic biosynthesis of biosurfactants. Three anaerobically producing strains of biosurfactants were *Pseudomonas stutzeri* Rhl, *P. aeruginosa* SG and *Bacillus subtilis* AnPL-1 (Zhao et al., 2015a,b, 2021). Three bacterial strains were incubated in liquid LB medium at 35°C for 16 h, respectively. The culture of three strains was serially diluted at tenfold gradient to 10^8^ times, respectively. The simulated experimental sample was prepared by mixing three diluted samples.

Here, 0.2 ml of simulated experimental sample were dispersed on solid LB medium plates. The anaerobic box was used for incubation of LB medium plates at 35°C. As shown in Table 1, 0.15 ml of fermentation medium was added into the 32 microwells at “column 3”, “column 6”, “column 9” and “column 12” of the 96-microwell plate. Then, 24 single strains isolated from LB plates were inoculated into microwells of “column 3”, “column 6” and “column 9”. The eight microwells on “column 12” were set as negative control without inoculum. The 96-microwell plates were also placed in anaerobic box. All the anaerobic culture broth in the 32 microwells was sampled to measure the diameters of oil displacement circles. The culture of 24 strains were inoculated into 20 ml anaerobic tubes containing 18 ml of fermentation medium using sterile syringe, respectively. Three parallels were set for each strain. Anaerobic medium was prepared as previously described (Javaheri et al., 1985; Zhao et al., 2015b, 2021). After culturing at 35°C, 80 rpm for 10 days, the surface tension of the anaerobic culture broth was measured. Strains reduced the surface tension to lower than 45 mN/m was verified for capable of anaerobic biosynthesis of biosurfactants.

**Table 1:** Microbial strains screened from the simulated environmental sample.

**Strain numbers**	**Diameter of oil displacement circle (mm)**	**Surface tension (mN/m)**	**Strain numbers**	**Diameter of oil displacement circle (mm)**	**Surface tension (mN/m)**
A3	13 ± 1.0	36.1 ± 0.7	E6	13 ± 0.5	37.1 ± 0.5
B3	13 ± 0.5	35.6 ± 0.6	F6	14 ± 1.0	36.4 ± 0.3
C3	17 ± 1.3	32.9 ± 0.4	G6	14 ± 0.5	35.8 ± 0.4
D3	14 ± 1.0	35.7 ± 0.7	H6	18 ± 0.5	32.4 ± 0.7
E3	13 ± 1.0	36.4 ± 0.8	A9	13 ± 1.0	36.3 ± 0.8
F3	12 ± 1.0	38.7 ± 0.7	B9	12 ± 0.5	36.3 ± 0.9
G3	13 ± 0.5	36.5 ± 0.4	C9	14 ± 0.5	35.7 ± 0.4
H3	13 ± 1.0	36.7 ± 0.8	D9	18 ± 0.5	31.9 ± 0.7
A6	14 ± 1.0	35.1 ± 0.7	E9	17 ± 1.0	33.2 ± 0.8
B6	16 ± 0.5	33.3 ± 0.7	F9	12 ± 0.5	38.9 ± 0.5
C6	13 ± 1.0	36.8 ± 0.3	G9	13 ± 1.0	36.2 ± 0.4
D6	14 ± 1.3	35.8 ± 0.7	H9	18 ± 1.0	32.7 ± 0.5

### Evaluation of the screening method using two environmental samples

In this study, two different kinds of environmental samples were used to evaluate the established screening method. Two environmental samples were oil reservoirs produced water and oily sludge. The sample of oily sludge (10 g) were added with 100 ml sterile water and 20 glass beads to shake at 25°Cand 200 rpm for 2 h to prepare suspension. The oil reservoirs produced water (0.20 ml) and oily sludge suspension (0.20 ml) were dispersed on solid LB medium plates, respectively. After incubation in the anaerobic box, single microbial colonies were then picked out. The selected strains were incubated one by one in the microwells of 96-microwell plates. After culturing at 35°C for 5 days in anaerobic box, strains whose culture broth in microwells can form oil displacement circles with diameters larger than 10 mm were screened as positive strains. The screened strains were incubated in anaerobic tubes at 35°C, 80 rpm for 10 days. The surface tension of anaerobic culture broth was measured to verify the strains function of anaerobic biosynthesis of biosurfactants. The screened microbial strains capable of anaerobic biosynthesis of biosurfactants were numbered, counted and used for further study.

### Identification of the screened strains

Some strains screened from two environmental samples were chosen for identification analysis by phylogenetic analysis of 16S rDNA sequence or ITS sequence. The genomic DNA of the selected strains was extracted by Genome DNA Extraction Kit DP302 (TIANGEN BIOTECH CO., LTD., Beijing, China). The 16S rRNA genes of strains were amplified using the universal PCR primers 27F (5-AGAGTTTGATCCTGGCTCAG-3) and 1492R (5-GGTTACCTTGTTACGACTT-3). The ITS genes of strains were amplified using the universal PCR primers ITS1 (5-TCCGTAGGTGAACCTGCGG-3) and ITS4 (5-TCCTCCGCTTATTGATATGC-3). The PCR products were sequenced by Sangon Biotech (Shanghai, China). The obtained 16S rDNA sequences were aligned using the online BLAST program (https://blast.ncbi.nlm.nih.gov/Blast.cgi) in the NCBI database. The software MEGA 5.0 was used to analyze the phylogenetic relationships of the obtained 16S rDNA sequences. The phylogenetic tree was constructed using Neighbor-Joining method at resampling bootstrap of 1,000.

### Analysis of influencing factors for the screening method

The potential influencing factors of the established screening method were also analyzed. The effect of enrichment culture treatment on the screening method was investigated using oil reservoir produced water as experimental samples. One sample of oil reservoir produced water (0.20 ml) was immediately dispersed on solid LB medium plates. Whereas another sample (8.0 ml) was firstly treated for anaerobic enrichment in 100 ml serum bottles containing 80 ml enrichment medium at 35°C for 6 days. The enrichment medium contained 30 g/l liquid paraffin, 3.0 g/l NaNO_3_, 3.0 g/l KH_2_PO_4_, 4.0 g/l K_2_HPO_4_·3H_2_O, 0.80 g/l MgSO_4_·7H_2_O, CuCl_2_ 0.1 g/l, CaCl_2_·2H_2_O 0.10 g/l, ZnCl_2_ 0.10 g/l, MnCl_2_·4H_2_O 0.10 g/l. The anaerobic enrichment culture was serially diluted at tenfold gradient to 10^8^ times. Then, 0.20 ml of diluted enrichment culture was dispersed on solid LB medium plates. Using the established screening method, single microbial colonies on the LB medium plates were numbered and classified based on colonial morphology. The number of single colonies and kinds of colonial morphology were used as indexes to evaluate the effect of enrichment culture treatment on the screening method.

Using oily sludge as experimental samples, the influence of sample suspension prepared by distilled water or normal saline was also studied. Two kinds of oily sludge suspension (0.20 ml) were dispersed on solid LB medium plates, respectively. Through the established screening method, single microbial colonies on the LB medium plates were numbered and classified based on colonial morphology. The number of single colonies and kinds of colonial morphology were used as indexes to evaluate the effect of distilled water and normal saline on the screening method.

## Results and discussion

### An efficient and simple screening method

As shown in Table 1, 24 single strains were screened from the simulated experimental sample which was a mixture of three previously obtained strains. The previously obtained strains *P. stutzeri* Rhl, *P. aeruginosa* SG and *B. subtilis* AnPL-1 are all microbial strains capable of anaerobic biosynthesis of biosurfactants (Zhao et al., 2015a,b, 2021). All the culture broth of 24 strains in the 96-microwells plate can form oil displacement circles with diameters greater than 10 mm. Whereas all the culture broth in microwells of “column 12” formed oil displacement circles with diameters smaller than 4 mm. After incubated in anaerobic tubes, all 24 strains reduced the surface tension of culture broth to lower than 40 mN/m. While the surface tension of culture broth in blank control group was 63.4 mN/m. The surface tension results confirmed that all screened 24 strains could produce biosurfactants under anaerobic conditions. Furthermore, the colonies of 24 strains on LB medium plates were divided into three categories, faint yellow and wet, green and wet, faint yellow and dry, which were exactly consistent with the colony morphology of the three used strains, *P. stutzeri* Rhl, *P. aeruginosa* SG and *B. subtilis* AnPL-1. It indicated that the 24 strains screened were derived from these three species of bacteria. Results confirmed that the screening method established in this study is accurate and reliable.

**Figure 1:**
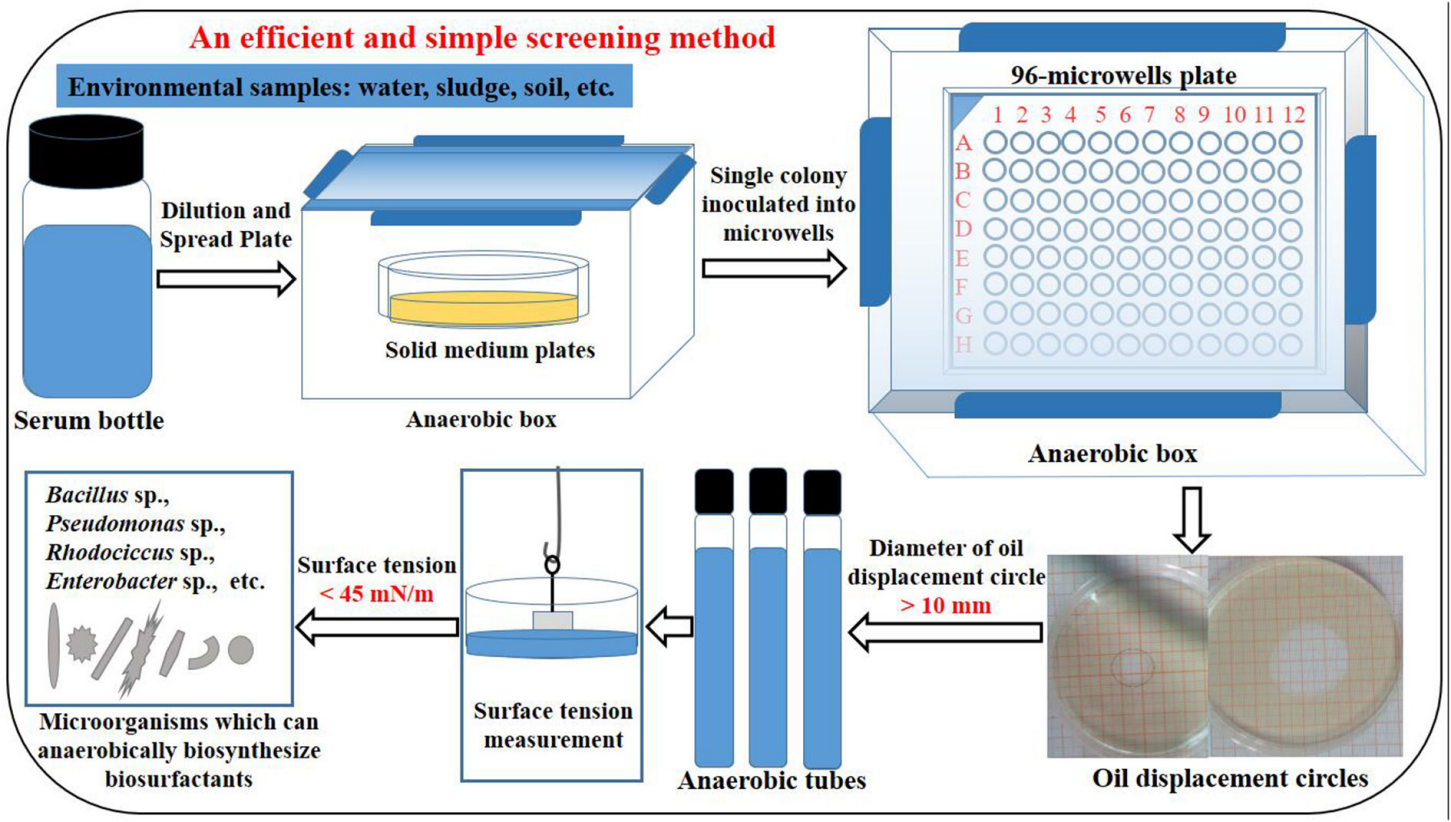
The procedure of an efficient and simple method for screening microbial strains capable of anaerobic biosynthesis of biosurfactants.

Using the previously obtained strains *P. stutzeri* Rhl, *P. aeruginosa* SG and *B. subtilis* AnPL-1, an efficient and simple method was established for screening microbial strains capable of anaerobic biosynthesis of biosurfactants. The procedure of established screening method was shown in Figure 1, and was briefly described as follow. This screening method included sample pretreatment, spread plate, anaerobic culture in 96-microwell plates, determining the oil displacement circle diameter of anaerobic culture broth, scale-up culture of screened strains in anaerobic tubes, and measuring the surface tension of anaerobic culture broth. Strains whose culture broth can form oil displacement circles with diameters larger than 10 mm were screened as positive strains. After culturing in anaerobic tubes, the positive strains were verified for capable of anaerobically biosynthesizing biosurfactants by reducing the surface tension of anaerobic culture broth to lower than 45 mN/m. With regards to the literature, there is no reports on special screening method for microbial resources capable of anaerobic biosynthesis of biosurfactants. This study reports an efficient and simple screening method. Besides, this established screening method did not require specialized chemicals and equipments, and produced few false positives.

### Evaluation of the established screening method

The established screening method were evaluated by two environmental samples. Here, 93 single colonies on LB medium plates were isolated from each environmental sample, oil reservoir produced water and oily sludge, respectively. The isolated 93 strains from each environmental sample were inoculated into the microwells of 96-microwells plates except for microwells of “A1”, “D6” and “H12”. The three microwells of “A1”, “D6” and “H12” were set as negative control without inoculum. As shown in Table 2, the culture broth of 21 strains in total screened from two 96-microwells plates can form oil displacement circles with diameters greater than 10 mm. Whereas all the culture broth in microwells of “A1”, “D6” and “H12” formed oil displacement circles with diameters smaller than 4 mm. Among the 21 strains, 8 strains were screened from the sample of oil reservoir produced water, and 13 strains were screened from the sample of oily sludge. After incubated in anaerobic tubes, all 21 strains reduced the surface tension of anaerobic culture broth to lower than 45 mN/m. While the surface tension of culture broth in blank control group was 63.4 mN/m. The surface tension results demonstrated that the 21 strains screened from two environmental samples factually produced biosurfactants under anaerobic conditions.

**Table 2:** The microbial strains screened from two environmental samples using the established method.

**Serial number**	**Strains**	**Diameter of oil displacement circle (mm)**	**Surface tension (mN/m)**
**Strains screened from the sample of oil reservoir produced water (“w” series)**			
1	wA4	10 ± 1.0	41.8 ± 0.6
2	wA5	13 ± 0.5	37.5 ± 0.6
3	wB3	13 ± 1.0	38.1 ± 0.2
4	wB11	14 ± 0.5	34.4 ± 0.4
5	wC10	12 ± 0.5	37.1 ± 0.4
6	wE11	13 ± 1.0	36.4 ± 0.8
7	wG6	12 ± 1.0	44.1 ± 0.6
8	wH3	15 ± 0.5	33.1 ± 0.9
**Strains screened from the sample of oily sludge (“s” series)**			
1	sA11	15 ± 0.5	32.4 ± 0.7
2	sB9	12 ± 0.5	37.3 ± 0.5
3	sC5	11 ± 0.5	40.7 ± 0.3
4	sC7	13 ± 1.0	37.8 ± 0.3
5	sD3	17 ± 1.0	32.7 ± 0.5
6	sD9	12 ± 0.5	39.2 ± 0.1
7	sD10	13 ± 1.0	38.6 ± 0.1
8	sE6	12 ± 1.0	39.1 ± 0.2
9	sF6	12 ± 0.5	40.7 ± 0.2
10	sF10	11 ± 1.0	43.3 ± 0.7
11	sG2	16 ± 0.5	33.4 ± 0.3
12	sG9	13 ± 1.0	38.1 ± 0.2
13	sH7	12 ± 0.5	38.5 ± 0.1

During the screening process, the oil displacement circle diameter of anaerobic culture broth in microwell is an accurate and reliable indicator for screening biosurfactants producing bacteria. The oil displacement circle diameter can also indirectly evaluate the yield of biosurfactants producing bacteria (Marchut-Mikołajczyk et al., 2021). There is a positive linear correlation between the diameters of oil spreading circle and the concentration of biosurfactants (Zhao et al., 2016; Jiang et al., 2020). Using the established method, all the strains screened from the samples of oil reservoir produced water and oily sludge can reduce the surface tension of anaerobic culture broth to lower than 45 mN/m. Results demonstrated that all screened strains can anaerobically produce biosurfactants. This screening method produced few false positives. This screening method can improve screening efficiency and screening flux, and laid a methodological foundation for developing more microbial resources capable of anaerobic biosynthesis of biosurfactants.

### Identification of the screened strains from two environmental samples

**Figure 2:**
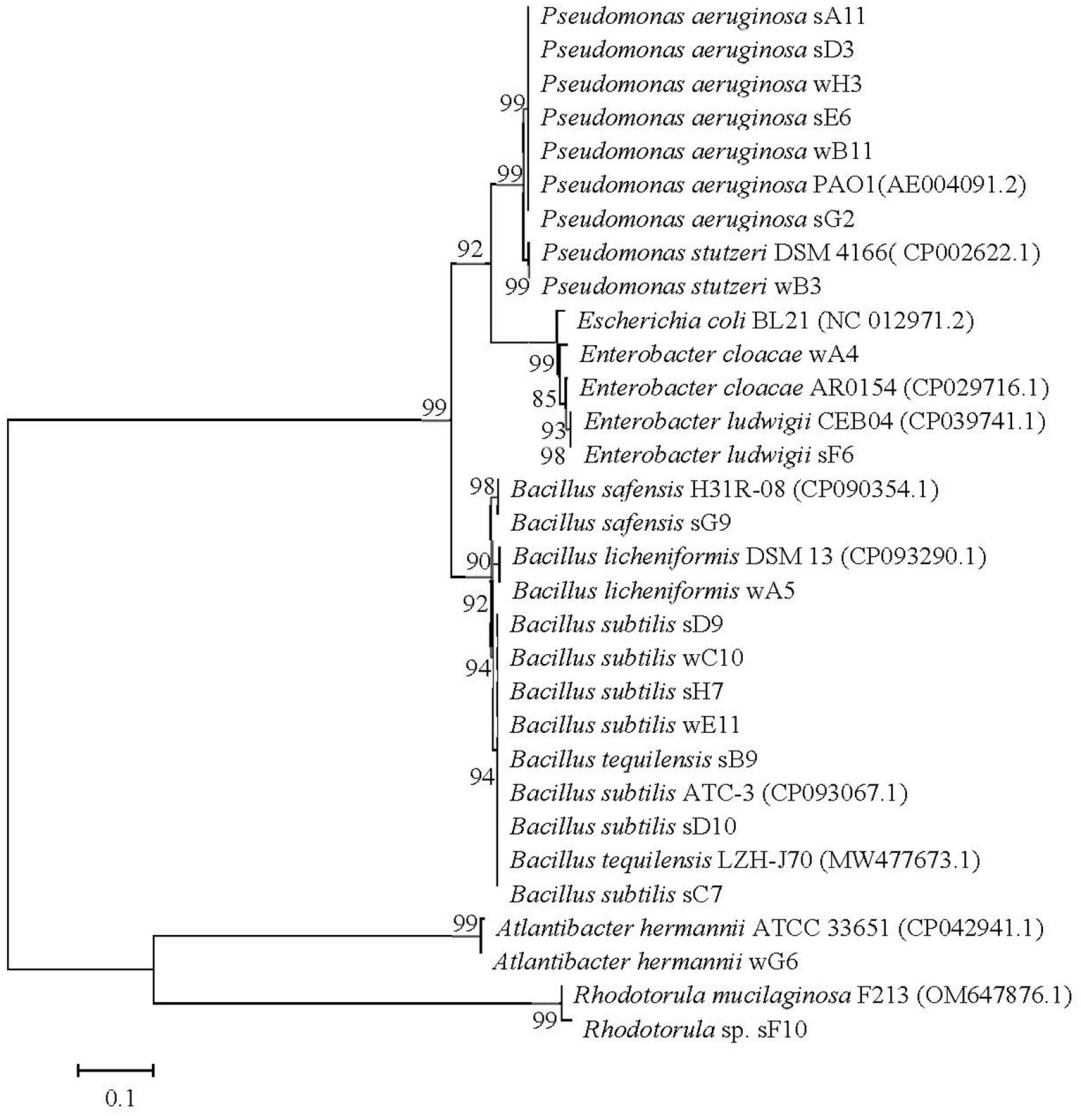
The phylogenetic tree of the screened microbial strains which can anaerobically biosynthesize biosurfactants, constructed by software Mega 5.0 based on a Neighbor-Joining Algorithm of 1000 resampled datasets. Bar, 0.1 nucleotide substitutions per site.

The screened microbial strains which can anaerobically biosynthesize biosurfactants were identified through the analysis of 16S rDNA or ITS sequences. Based on the obtained 16S rDNA or ITS sequences, the constructed phylogenetic tree as shown in Figure 2. Numbers at nodes indicate levels of bootstrap support (%) based on a neighbor-joining analysis of 1,000 resampled datasets. All the values at nodes are higher than 85% in the phylogenetic tree. Through phylogenetic analysis, the microbial strains screened from oil reservoir produced water were identified as *Pseudomonas* sp., *Bacillus* sp., *Escherichia hermannii*, and *Enterobacter* sp. The microbial strains screened from oily sludge were identified as *P. aeruginosa, Bacillus* sp., *Aspergillus* sp., and *Enterobacter* sp. Studies reported that biosurfactants were generally biosynthesized by some strains of bacteria, yeast and fungi (Kashif et al., 2022). The reported biosurfactants producing strains are *Pseudomonas* sp., *Burkholderia* sp., *Starmerella* sp., *Candida* sp., *Bacillus* sp., *Acinetobacter* sp., *Rhodococcus* sp., *Ochrobactrum* sp., *Rhodociccus* sp., *Aspergillus* sp., *Klebsiella* sp., etc. (Bernat et al., 2019; Fenibo et al., 2019; Markande et al., 2021; Sarubbo et al., 2022).

### Potential influencing factors of the established screening method

The effect of enrichment culture treatment was investigated using samples of oil reservoir produced water. Through the established screening method, the number of single colonies and kinds of colonial morphology on the LB medium plates were used as evaluation indicators. Here, average 273 single microbial colonies and 9 kinds of colonies were obtained by enrichment culture before screening, and average 139 microbial colonies and 17 kinds of colonies were obtained without enrichment culture treatment before screening (*P* = 0.03 < 0.05). Results showed that more microbial strains might be isolated after enrichment of the environmental samples. Whereas more variety of microbial strains would be isolated without enrichment of the environmental samples before screening.

Taking oily sludge as experimental samples, the influence of distilled water or normal saline to prepare sample suspension was studied. The number of single colonies and kinds of colonial morphology on the LB medium plates were also used as indexes to evaluate the effect of distilled water and normal saline on the screening method. Here, average 79 microbial colonies and 9 kinds of colonies were obtained when the oily sludge suspension prepared with distilled water, and average 85 microbial colonies and 9 kinds of colonies were obtained when the oily sludge suspension prepared with normal saline (*P* = 0.464 > 0.05). In terms of colony numbers and colonial morphology, sample suspension prepared with distilled water or normal saline had no significant effect on the screening method.

### Prospects and perspectives

Biosurfactants exhibit excellent performance, great application potential and incomparable advantages against chemical surfactants (Badmus et al., 2021; Johnson et al., 2021). With increasing awareness of environmental protection, more and more attention has been paid to biosurfactants. Biosurfactants producing bacteria are essential for applications of biosurfactants (Rawat et al., 2020; Sarubbo et al., 2022). The application of biosurfactants can be divided into the microbial *in-situ* production of biosurfactants in environments and the microbial production of biosurfactants in fermenters. Therefore, development of highly adaptable microbial resources and the breeding of high-yield microbial strains are the main research interests of biosurfactants producing bacteria (Perfumo et al., 2018; Tripathi et al., 2018; Silva et al., 2019). For some oxygen-deficient environments, the microbial strains which can anaerobically biosynthesize biosurfactants are needed (Domingues et al., 2017; Li et al., 2021).

In this study, an efficient and simple method was established for screening microbial strains capable of anaerobic biosynthesis of biosurfactants, based on the oil displacement characteristic of biosurfactants combined with the anaerobic culture technology using microplate assays. After evaluation by two environmental samples, oil reservoirs produced water and oily sludge, the target microbial strains screened by this method showed efficient and low false positive. Results revealed that the oil displacement circle diameter of anaerobic culture broth is an accurate and reliable indicator for screening microbial strains capable of anaerobic biosynthesis of biosurfactants. This established screening method is of strong specificity, less sample consumption and high experimental flux.

Although there are many microbial secondary metabolites that have activity to reduce surface tension, these microbial secondary metabolites can also be referred to as biosurfactants (Bhadra et al., 2022). Some new biosurfactants other than rhamnolipid and lipopeptide may be obtained from these screened microbial strains. This microbial screening method cannot differentiate the types of produced biosurfactants. In the future, the types of the produced biosurfactants will be further studied.

Although 186 microbial colonies were screened from two environmental samples, there were relatively few in number and single in species of microorganisms which can anaerobically biosynthesize biosurfactants. This study also found that when liquid paraffin was used as carbon source and nitrate was used as nitrogen source before screening, the number of single colonies was increased, but the kinds of colonial morphology was relatively simple. In the future, selecting suitable carbon and nitrogen sources and designing suitable enrichment medium would be beneficial to screen biosurfactant producing bacteria with special metabolic activity. Besides, the isolation of microorganisms from diverse sources, including contaminated environmental samples, sediments, food waste or agro-industrial wastes would be an important approach to screen more adaptable microbial strains for biosurfactants production (Tripathi et al., 2018; Pessôa et al., 2019).

## Conclusions

An efficient and simple method was established for screening microbial strains capable of anaerobic biosynthesis of biosurfactants, based on the oil displacement characteristic of biosurfactants combined with the anaerobic culture technology using microplate assays. Using this method, microbial strains which can anaerobically biosynthesize biosurfactants were successfully screened from reservoir produced water and oily sludge, respectively. The diameter of oil displacement circle is a reliable screening indicator. This established screening method is highly targeted, low false positive, easy to implement and efficient. More microbial strains might be isolated after enrichment of the environmental samples, while more variety of microbial strains would be isolated without enrichment. This established screening method laid a methodological foundation to investigate microbial resources which can anaerobically biosynthesize biosurfactants from environmental samples.

## Data availability statement

The raw sequencing data of 16S rDNA were deposited in the GenBank database of National Center for Biotechnology Information with accession numbers from OP060719 to OP060737. The raw sequencing data of ITS were deposited in the GenBank database of National Center for Biotechnology Information with accession numbers OP060798 and OP060799.

## Author contributions

FZ: conceptualization, methodology, investigation, writing, supervision, project administration, and funding acquisition. YW: investigation, validation, writing, and visualization. XHu: investigation, validation, and formal analysis. XHuang: investigation, formal analysis, and visualization. All authors contributed to the article and approved the submitted version.

## Funding

This work was financially supported by the Research Start-Up Foundation for Introduced Talent of Qufu Normal University (6096).

## Conflict of interest

The authors declare that the research was conducted in the absence of any commercial or financial relationships that could be construed as a potential conflict of interest.

## Publisher's note

All claims expressed in this article are solely those of the authors and do not necessarily represent those of their affiliated organizations, or those of the publisher, the editors and the reviewers. Any product that may be evaluated in this article, or claim that may be made by its manufacturer, is not guaranteed or endorsed by the publisher.
